# Compressive Sensing Based Multilevel Fast Multipole Acceleration for Fast Scattering Center Extraction and ISAR Imaging

**DOI:** 10.3390/s18072024

**Published:** 2018-06-25

**Authors:** Wei Zhu, Ming Jiang, Xin He, Jun Hu

**Affiliations:** School of Electronic Science and Engineering, University of Electronic Science and Technology of China, Chengdu 611731, China; happyweiwei@uestc.edu.cn (W.Z.); jiangming@uestc.edu.cn (M.J.); he.xin.1987@163.com (X.H.)

**Keywords:** multilevel fast multipole acceleration (MLFMA), compressive sensing (CS), electromagnetic (EM), inversed synthetic aperture radar (ISAR), Fast Fourier Transform (FFT)

## Abstract

In recent years, Compressive Sensing (CS) theory has been very popular in the data sensing and process area as it utilizes the sparsity and measurement matrix to reconstruct the compressible signal from limited samples successfully. In this paper, CS is introduced into an efficient numerical method, multilevel fast multipole acceleration (MLFMA), for the electromagnetic (EM) scattering problem over a wide incident angle. This allows composition of a new kind of incident wave, which obtains efficient and reliable data for scattering centers extraction with low complexity. The resulting data from CS-based MLFMA are processed for ISAR) imaging. Simulation results show the received data for ISAR imaging from MLFMA with CS can outperform the data from MLFMA, which achieves a similar quality of ISAR imaging. Additionally, the computation complexity is improved by CS through the reduced matrix computation for fewer incident waves. It makes ISAR imaging using real data feasible and meaningful.

## 1. Introduction

In recent years, the Inverse Synthetic Aperture Radar (ISAR) technique for the imaging of moving targets has attracted more and more attention to investigate the scattering mechanism of complex targets and target identification [1]. Based on the received signal after compensation, the traditional method is Range-Doppler (RD) for ISAR imaging in the range and Doppler (cross-range) directions [2]. The target can be imaged by using a two-dimensional Fast Fourier Transform Algorithm (2D-FFT) for the received signal. The range resolution is decided by the signal bandwidth, while the cross-range resolution is decided by the wavelength and the range of observation, so that certain rotation angles of the target with respect to the radar line of sight during the coherent processing interval (CPI) are required for the high cross-range resolution [2].

To predict the radar signal of the target accurately, it is necessary to study the full wave electromagnetic (EM) scattering mechanism of the complex target. In real-life applications, due to the high economic cost and the complex field measurements of multiple scattering effects, EM computation is an effective and economical way to simulate the scattering echoes of targets for ISAR imaging research [3,4]. The numerical methods to solve fast simulation of EM scattering problems over a wide angle can be classified into two groups: integral equations and differential equations. The super-resolution methods Multiple Signal Classification (MUISC) and Estimation Signal Parameters via Rotational Invariance Techniques (ESPRIT) are designed based on the differential equation Finite-Difference Time-Domain (FDTD) to obtain high quality imaging with lower computation complexity [3]. On the other hand, the traditional integral equations, such as the method of moments (MoM) [5,6] and the finite element method (FEM) [7,8], can also be used to obtain the received data for ISAR imaging [4]. These methods need to repeat the solution of the system matrix equation using the iteration method in every incident angle. A variety of fast algorithms, such as multilevel fast multipole acceleration (MLFMA), the adaptive integral method (AIM), and pre-corrected FFT (PC-FFT) have been developed to greatly reduce the computational cost [9,10] for the analysis of electrically large objects, however, the accelerated solution process is still inefficient.

As discussed above, many measurements of massive data and huge computation are needed for ISAR imaging with EM, which cannot be achieved in the real world due to the expensive equipment or cost. Fortunately, the concept of compressive sensing (CS) that was proposed by Candes in 2006 [11,12] is based on the intrinsically or extrinsically sparse signals that can be represented by nonzero expansion coefficients and the corresponding expansion base [13,14,15,16]. CS theory recovers the signals with far fewer samples, a great breakthrough of the common Nyquist–Shannon’s sampling theorem. CS theory has also developed the new research field of EM [17,18,19], which includes antenna arrays, inverse scattering, and radar imaging [20,21,22,23,24,25], and it is also expected to be used for data processing in computing networks [26] in the future, such as in edge computing [27,28,29,30]. Sparsity and incoherence are the two key principles in CS theory. CS pertains to exploit a priori information (sparsity with respect to a basis) and few (incoherent) measurements for retrieving unknown signals, which turns the sampling of signal into the sampling of information. The signals with sparsity are recovered by solving a $l_{1}$-norm optimization problem [31]. In other words, the ill-posed problem, recovering high-dimensional signal from low-dimensional observations, could be solved by exploiting sparsity of the objective signal [32].

For monostatic EM scattering problems over a wide angle, the CS-based technique is introduced into MLFMA (CS-MLFMA) in this paper to get a new incentives incidence model, with which the number of the incidents angles are reduced, and the compressed data can be obtained to extract the independently distributed function of the scattering centers for ISAR imaging. Section 2 presents the analysis of the CS theory and the recovery process of the sparse signal. In addition, the MLFMA for a wide-angle EM scattering problem is described in Section 3, and it is also proposed how the CS theory implemented in MLMFA obtains the received data, which are then exploited to recover the images in ISAR imaging by FFT. Section 4 presents the experimental results of CS-MLFMA compared with MLFMA, as well as the imaging results from the two methods, respectively. Computation complexity and computing time comparison are practically detailed in this section. Finally, conclusions are drawn in Section 5.

## 2. Compressive Sensing

The three key points of the popular CS theory are the sparsity of the received signals, the incoherent measurement matrix, and the robust reconstruction algorithm [14].

Let us consider a signal x ∈ $R^{N}$ with length of N, which can be interpreted as an N column vector and its sparsity is K, which means there are *K* ≪ *N* nonzero coefficients at most. That is to say, at most, K rather than N dimensions of information exist in the signal x, so that x can be expressed as:

(1) x=Ψa

where a is the N×1 column vector of weighting coefficients $a_{i}=\langle x_{i},\psi_{i}\rangle =\psi_{i}^{T}x$ corresponding to the basis column vector $\psi_{i}$ of the basis matrix $\Psi =\left\{\psi_{1},\psi_{2},\ldots ,\psi_{N}\right\}$. ${\left(.\right)}^{T}$ denotes the transpose operations.

In terms of signal acquisition, by using an M×N sensing matrix Φ, the signal can be measured from N dimensions reducing into K∼M dimensions, where the measuring times M should be as follows:

(2) $$M=O\left(Klog_{2}\left(\frac{N}{K}\right)\right)$$

The signal can be obtained as:

(3) y=Φx=ΦΨa=Aa

where A = ΦΨ is an M×N measurement matrix which should satisfy the restricted isometry property (RIP) [31,33] of order K with constant 0<δ<1 if, for all *K*-sparse $x\in R^{N}$, the following condition is true:

(4) $$1-\delta \leq ∥\frac{Ax{∥}_{P}}{∥x{∥}_{l_{P}}}\leq 1+\delta$$

with $∥.{∥}_{l_{P}}$ being the $l_{P}$-norm. The equivalent condition of RIP is that the measurement matrix Φ is incoherent with the basis matrix Ψ.

The reconstruction of x from y can be obtained with high probability via the $L_{1}$ norm minimization as below:

(5) $$\hat{a}=min∥a{∥}_{L_{1}}\;subject\;to\;y=Aa$$

The original signal can be estimated as:

$$\hat{x}=\Psi \hat{a}$$

## 3. Implementation of CS in MLFMA

### 3.1. MLFMA

For the surface of the ideal conducting object, the electric field integral equation (EFIE) and magnetic field integral equation (MFIE) can be expressed respectively as follows:

(6) $$\hat{t}\times \vec{E^{inc}\left(\vec{r}\right)}=\hat{t}\times \left(j\omega \vec{J_{s}\left(\vec{r}\right)}+\nabla \Phi_{s}\left(\vec{r}\right)\right)$$

(7) $$\hat{n}\times \vec{H^{inc}\left(\vec{r}\right)}=\vec{J_{s\left(\vec{r}\right)}}\;-\hat{n}\times \nabla \Phi_{s}\left(\vec{r}\right)$$

where $\vec{E^{inc}\left(\vec{r}\right)}$ and $\vec{H^{inc}\left(\vec{r}\right)}$ are the electric and magnetic field intensity of the incident wave, respectively, ω is the angular frequency, $\vec{J_{s}}$ is the magnetic vector potential, $\Phi_{s}$ is electric scalar potential, $\hat{n}$, $\vec{r}$ and j stands for normal direction, field point, and imaginary part, respectively.

It is well-known that the EFIE is suitable for any open and closed conducting objects thanks to its high-precision solution, however, due to its slow rate of convergence MFIE is added to implement the coupled field integral equation (CFIE) [7]:

(8) $$\begin{array}{c} CFIE=\alpha EFIE+\left(1-\alpha \right)Z_{0}\times MFIE \end{array}$$

with α∈[0,1] and $Z_{0}=\sqrt{\frac{\varepsilon_{0}}{\mu_{0}}}$ is the intrinsic impedance of space.

After using basis and testing functions in Equations (6) and (7), we write a N×N dense matrix equation as follows:

(9) ZX=V

where N represents the number of unknowns for electric currents. Z represents the MoM impedance matrix and X represents the unknown coefficient vector to be determined. V is referred to as the excitation vector. The resultant dense matrix equation is very expensive both in terms of computational cost and memory storage. The resultant matrix equation is directly solved by employing an iterative method based on a Krylov subspace algorithm (for example, the generalized minimal residual (GMRES), conjugate gradient (CG) method.), requiring $O\left(N^{2}\right)$ complexity for both the matrix-vector multiplies and memory. The required matrix-vector multiplications (MVM) are performed efficiently with MLFMA, which reduces both storage complexity and computational complexity into O(NlogN). MVM are divided into two parts by MLFMA in the manner of groups: near interactions and far interactions. Generally, when the distance between radiating groups and receiving groups are no more than half a wavelength away, they are considered near interactions, otherwise they are far interactions. The former is computed by MoM directly, while the latter can be accelerated by MLFMA. The basic idea of MLFMA is to separate the interaction into three steps: aggregation, translation, and disaggregation. Firstly, radiated fields of each group are obtained from the finest level of the oct-tree structure to the highest level. The contribution of radiated field of basis functions in the finest level is aggregated into the center of each group. In the upper levels, the radiated field of one parent group is the combination of the radiated fields of its son-groups. In the translation step, radiated fields computed during the aggregation step are translated into incoming fields. Incoming fields at group centers are then distributed from the highest level to the finest level in the disaggregation step. In other words, the total incoming field for one group is obtained by combining incoming fields due to translations and the incoming field to the center of its parent group. The details of MLFMA has been investigated extensively in various references [9,10].

### 3.2. CS-MLFMA

Using the MoM, after discretization and testing, Equation (9) can be rewritten as below:

(10) $$\begin{array}{c} \overset{N}{\underset{i=1}{\sum }}Z_{ji}x_{i}=v_{j},\;j=1,\;2,\ldots ,N \end{array}$$

where $Z_{ji}$ means the impendence matrix Z only related to the incident frequency, v is the incident wave and x is the current coefficient. Equation (10) can be expressed as:

(11) $$\begin{array}{c} Z\left(f\right)X\left(\theta ,f\right)=V\left(\theta ,f\right)\; \end{array}$$

where θ is the incident angle, f is the incident frequency, V is the excitation vector related to the incident angle θ and the incident frequency f, and X is the current coefficient vector to be solved. Assuming f as a fixed value $f_{0}$, incident angles are $\theta_{1},\theta_{2},\ldots ,\;\theta_{n}$, Equation (11) can be rewritten as:

(12) $$\begin{array}{c} Z\left(f_{0}\right)\left[X\left(\theta_{1},f_{0}\right),X\left(\theta_{2},f_{0}\right),\ldots ,X\left(\theta_{n},f_{0}\right)\right]=\left[V\left(\theta_{1},f_{0}\right),V\left(\theta_{2},f_{0}\right),\ldots ,\;V\left(\theta_{n},f_{0}\right)\right] \end{array}$$

Equation (12) can be transformed into Equation (13) by taking the transpose of Z, X and V.

(13) $$\begin{array}{c} \left[\begin{array}{c} \begin{array}{c} X^{T}\left(\theta_{1},f_{0}\right)\\ X^{T}\left(\theta_{2},f_{0}\right)\\ \ldots \end{array}\;\\ X^{T}\left(\theta_{n},f_{0}\right) \end{array}\right]Z^{T}\left(f_{0}\right)=\left[\begin{array}{c} \begin{array}{c} V^{T}\left(\theta_{1},f_{0}\right)\\ V^{T}\left(\theta_{2},f_{0}\right)\\ \ldots \end{array}\\ V^{T}\left(\theta_{n},f_{0}\right) \end{array}\right] \end{array}$$

For the monostatic radar cross section (RCS) EM scattering over a wide angle, the computation of solving the matrix equation at each incident angle is very time-consuming. Additionally, there is a requirement to fill in the matrix with the changed angle as the incident angles increase and then solve the equation. Therefore, the computation complexity is huge. Thanks to CS, Equation (13) can be described as Equation (14) with the sparse representation as in Equation (1).

(14) $$\begin{array}{c} \Psi_{n\times n}\left[\begin{array}{c} \begin{array}{c} a\left(\theta_{1},f_{0}\right)\\ a\left(\theta_{2},f_{0}\right)\\ \ldots \end{array}\\ a\left(\theta_{n},f_{0}\right) \end{array}\right]Z^{T}\left(f_{0}\right)=\left[\begin{array}{c} \begin{array}{c} V^{T}\left(\theta_{1},f_{0}\right)\\ V^{T}\left(\theta_{2},f_{0}\right)\\ \ldots \end{array}\\ V^{T}\left(\theta_{n},f_{0}\right) \end{array}\right] \end{array}$$

In other words, the incident waves can be viewed as a new kind of incident wave by adding the incident waves from different angles together randomly as below:

(15) $$V^{CS}=\overset{N}{\underset{i=1}{\sum }}o_{i}V^{T}\left(\theta_{i},f_{0}\right)$$

where $o_{i}$ is the coefficient of each incident wave. Because of the invariable Z related to the incident angle, the induced current invoked by the new kind of incident wave is equivalent to the sum of the separately induced current by each incident wave from each angle as:

(16) $$X^{CS}=\overset{N}{\underset{i=1}{\sum }}o_{i}X^{T}\left(\theta_{i},f_{0}\right)$$

At different angles, the $\theta_{i}$ current based on some basis changes regularly. Therefore, as for some basis on the surface of the scattering center, CS theory can be implemented to reduce the number of the incidence from the original n times to m times by linear combination. The m times of the induced current can be described as:

(17) $$\begin{array}{c} \left[\begin{array}{c} \begin{array}{c} X^{CS}\left(\theta_{1},f_{0}\right)\\ X^{CS}\left(\theta_{2},f_{0}\right)\\ \ldots \end{array}\;\\ \;X^{CS}\left(\theta_{m},f_{0}\right) \end{array}\right]=\phi_{m\times n}\left[\begin{array}{c} \begin{array}{c} X^{T}\left(\theta_{1},f_{0}\right)\\ X^{T}\left(\theta_{2},f_{0}\right)\\ \ldots \end{array}\;\\ \;X^{T}\left(\theta_{n},f_{0}\right) \end{array}\right] \end{array}$$

where the matrix ϕ can be viewed as the measurement matrix in the CS theory:

(18) $$\begin{array}{c} \phi_{m\times n}=\left[\begin{array}{ccc} o_{11} & \cdots & o_{1n}\\ \vdots & \ddots & \vdots \\ o_{m1} & \cdots & o_{mn} \end{array}\right] \end{array}$$

Adding the measurement matrix in Equation (14), the equation is as follows:

(19) $$\begin{array}{c} \phi_{m\times n}\Psi_{n\times n}\left[\begin{array}{c} \begin{array}{c} a\left(\theta_{1},f_{0}\right)\\ a\left(\theta_{2},f_{0}\right)\\ \ldots \end{array}\;\\ \;a\left(\theta_{n},f_{0}\right) \end{array}\right]Z^{T}\left(f_{0}\right)=\phi_{m\times n}\left[\begin{array}{c} \begin{array}{c} V^{T}\left(\theta_{1},f_{0}\right)\\ V^{T}\left(\theta_{2},f_{0}\right)\\ \ldots \end{array}\;\\ \;V^{T}\left(\theta_{n},f_{0}\right) \end{array}\right] \end{array}$$

As discussed above, the reconstructed signal can be obtained by

(20) $$\begin{array}{c} \left[\begin{array}{c} \begin{array}{c} X^{T}\left(\theta_{1},f_{0}\right)\\ X^{T}\left(\theta_{2},f_{0}\right)\\ \ldots \end{array}\;\\ \;X^{T}\left(\theta_{n},f_{0}\right) \end{array}\right]=\Psi_{n\times n}\left[\begin{array}{c} \begin{array}{c} \hat{a}\left(\theta_{1},f_{0}\right)\\ \hat{a}\left(\theta_{2},f_{0}\right)\\ \ldots \end{array}\;\\ \;\hat{a}\left(\theta_{n},f_{0}\right) \end{array}\right] \end{array}$$

where $\hat{a}\left(\theta_{i},f_{0}\right)$(i = 1, 2, 3, …, n) can be estimated from Equation (5). Therefore, the new incident waves can be constructed as follows:

(21) $$\begin{array}{c} \left[\begin{array}{c} \begin{array}{c} X^{CS}\left(\theta_{1},f_{0}\right)\\ X^{CS}\left(\theta_{2},f_{0}\right)\\ \ldots \end{array}\;\\ \;X^{CS}\left(\theta_{m},f_{0}\right) \end{array}\right]=\phi_{m\times n}\left[\begin{array}{c} \begin{array}{c} X^{T}\left(\theta_{1},f_{0}\right)\\ X^{T}\left(\theta_{2},f_{0}\right)\\ \ldots \end{array}\;\\ X^{T}\left(\theta_{n},f_{0}\right) \end{array}\right] \end{array}$$

Taking Equation (19) into Equation (20), the new incident waves can be expressed as:

(22) $$\begin{array}{c} \left[\begin{array}{c} \begin{array}{c} X^{CS}\left(\theta_{1},f_{0}\right)\\ X^{CS}\left(\theta_{2},f_{0}\right)\\ \ldots \end{array}\;\\ \;X^{CS}\left(\theta_{m},f_{0}\right) \end{array}\right]=\phi_{m\times n}\Psi_{n\times n}\left[\begin{array}{c} \begin{array}{c} \hat{a}\left(\theta_{1},f_{0}\right)\\ \hat{a}\left(\theta_{2},f_{0}\right)\\ \ldots \end{array}\;\\ \;\hat{a}\left(\theta_{n},f_{0}\right) \end{array}\right] \end{array}$$

From Equation (22), it is known that the new constructed induced current by CS, which is equivalent to implementing the compressing and measuring processes at the same time, can be obtained by reconstructing the m(m≈Klog(n/K)≪n) measurements, where K is the sparsity of the induced current signal. In this way, the problem of the incident waves from n single angles is changed into the problem of the incident waves from the combined angles. Usually, the measurement matrix is set to Gauss random matrix which satisfies the RIP but increases the advantages of reducing the computation complexity by implementing CS theory in EM due to the increased computation for the full-rank measurement matrix in the reconstruction process. Therefore, the measurement matrix is constructed as in Equation (23), which should not only satisfy RIP but also needs to void the recycling computation for each incidence by the linear combination of incident waves without repeated information but with completive information [34]:

(23) $$\begin{array}{c} \phi_{m\times n}={\left[\begin{array}{ccc} o_{11} & \cdots & 0\\ \vdots & \ddots & \vdots \\ 0 & \cdots & o_{mm} \end{array}\begin{array}{ccc} o_{1,m+1} & \cdots & 0\\ \vdots & \ddots & \vdots \\ 0 & \cdots & o_{m,2m} \end{array}\begin{array}{ccc} o_{1,j} & \cdots & 0\\ \vdots & \ddots & \vdots \\ 0 & \cdots & o_{m,n} \end{array}\right]}_{m\times n} \end{array}$$

Obviously, the new constructed measurement matrix with sparsity reduces the times of multiplication in Equation (23) from m×n into n times, so that the estimated signal is simplified, and the recovery of reconstruction is accelerated.

### 3.3. ISAR Imaging by CS-MLFMA

**Figure 1:**
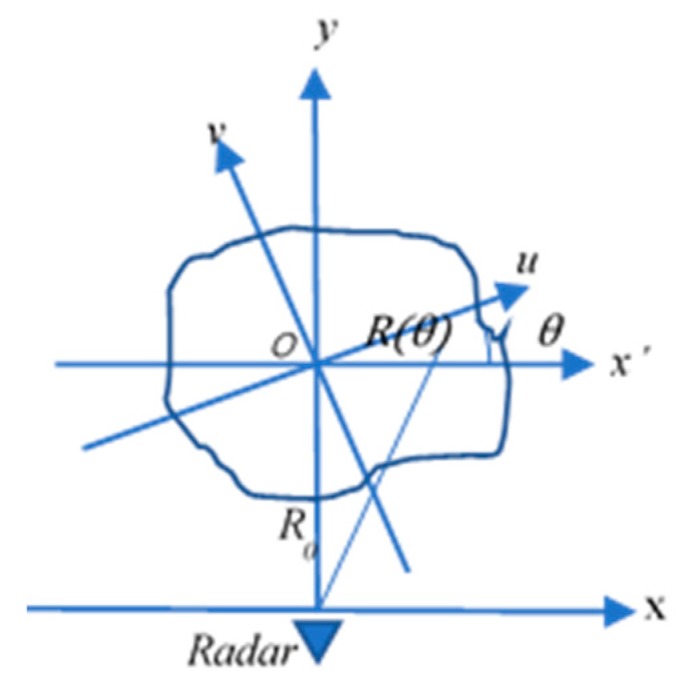
Inverse Synthetic Aperture Radar (ISAR) Imaging Model.

ISAR imaging can be viewed as turntable imaging after the received signal is processed by motion compensation. The target on the turntable turns a very small angle θ during the twice-observed duration, as shown in Figure 1, where $R_{0}$ is the distance between the horizontal axis-X and axis-X’. R(θ), the distance from the observed point on the target to the radar, is expressed as:

(24) $$\begin{array}{c} R\left(\theta \right)=\sqrt{x_{1}{}^{2}+\left(y_{1}{}^{2}+\;R_{0}{}^{2}\right)}\; \end{array}$$

The relationships among the coordinates are described as below:

(25) $$\begin{array}{c} \{\begin{array}{c} x=x_{1}\\ y=y_{1}+r_{0} \end{array} \end{array}$$

(26) $$\begin{array}{c} \{\begin{array}{c} x_{1}=u\cos\theta +v\sin\theta \\ y_{1}=v\cos\theta -u\sin\theta \end{array}\; \end{array}$$

Using Equations (25) and (26), Equation (24) can be expressed as in Equation (27) and be approximated as Equation (28) if $r_{0}$ is very far from the target size.

(27) $$\begin{array}{c} R\left(\theta \right)=\sqrt{r_{0}{}^{2}+u^{2}+\;v^{2}+2R_{0}v\cos\theta -2R_{0}u\sin\theta } \end{array}$$

(28) $$\begin{array}{c} R\left(\theta \right)\approx R_{0}+v\cos\theta -u\sin\theta \end{array}$$

Assuming the transmitted signal of the radar is $s_{t}=e^{j2\pi ft}$, where f is the frequency, the received data can be obtained as in Equation (29), where *c* is the light velocity and g(u,v) is 2-D distribution function of the target.

(29) $$\begin{array}{c} X\left(\theta ,t\right)=\iint g\left(u,v\right)e^{j2\pi f\left[t-\frac{2R\left(\theta \right)}{c}\right]}dudv\; \end{array}$$

When the scene of the target is in the far field, Equation (29) can be simplified as:

(30) $$\begin{array}{c} X\left(\theta ,t\right)=\iint g\left(u,v\right)e^{j2\pi ft}e^{-j\frac{4\pi f}{c}\left(R_{0}+v\cos\theta -u\sin\theta \right)}dudv \end{array}$$

In the frequency domain, the received signal can be transformed by FFT as in Equation (31).

(31) $$\begin{array}{c} X\left(\theta ,f\right)=\iint g\left(u,v\right)e^{-j\frac{4\pi f}{c}\left(R_{0}+v\cos\theta -u\sin\theta \right)}dudv \end{array}$$

Proved by multiple scattering centers, the EM scattering field of the target can be equivalent to the superposition of the strong scattering points in a high frequency area.

(32) $$\begin{array}{c} X\left(\theta ,f\right)=\overset{P}{\underset{p=1}{\sum }}\sigma_{p}e^{-j\frac{4\pi f}{c}\left(R_{0}+v\cos\theta -u\sin\theta \right)}\; \end{array}$$

where $\sigma_{p}$ is the $p_{th}$ backscattering coefficients in the field of angle θ.

If there is a group of impulses with $N^{f}$ frequencies stepping uniformly while in the angle $\theta_{N^{\theta }}$ duration, the received data can be constructed as in Equation (33).

(33) $$\begin{array}{c} X\left(\theta ,f\right)=\left[\begin{array}{ccc} X\left(\theta_{1},f_{1}\right) & \cdots & X\left(\theta_{1},f_{N^{f}}\right)\\ \vdots & \ddots & \vdots \\ X\left(\theta_{N^{\theta }},f_{1}\right) & \cdots & X\left(\theta_{N^{\theta }},f_{N^{f}}\right) \end{array}\right] \end{array}$$

Therefore, the distribution function of the scattering center g(u,v) can be retrieved from Equation (28) by FFT for ISAR imaging as in Equation (34).

(34) $$\begin{array}{c} g\left(u,v\right)=\iint X\left(\theta ,f\right)e^{-j\frac{4\pi f}{c}\left(R_{0}+v\cos\theta -u\sin\theta \right)}dudv \end{array}$$

## 4. Numerical Examples 

To evaluate the effectiveness of the CS theory used in EM, some common models are experimented with by utilizing the proposed methods of CS-MLFMA. The RCS data are then used for ISAR imaging, which proves that the proposed method in Section 3 can recover the received data successfully.

### 4.1. CS-MLFMA

**Figure 2:**
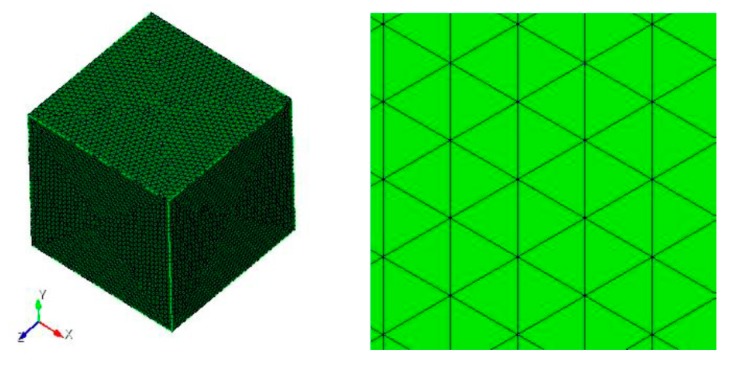
PEC cube model.

As mentioned in Section 2, a Perfect Electric Conductor (PEC) cube with the side length of 3 m shown in Figure 2 is taken into consideration and analyzed.

**Figure 3:**
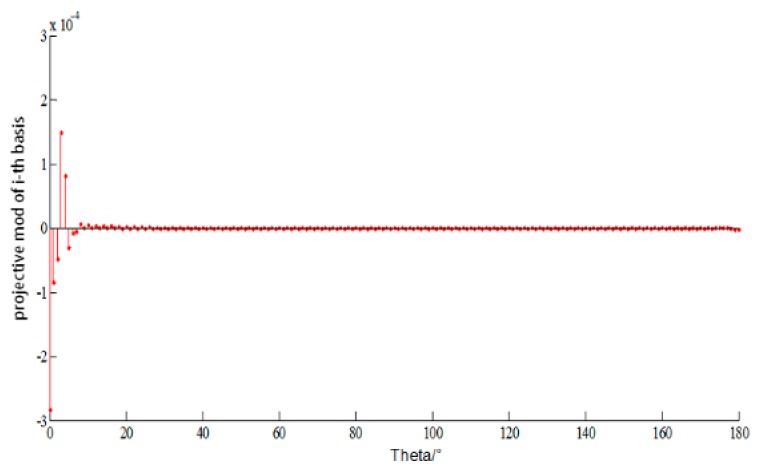
The projection value of the original current of PEC cube based on DCT basis.

**Figure 4:**
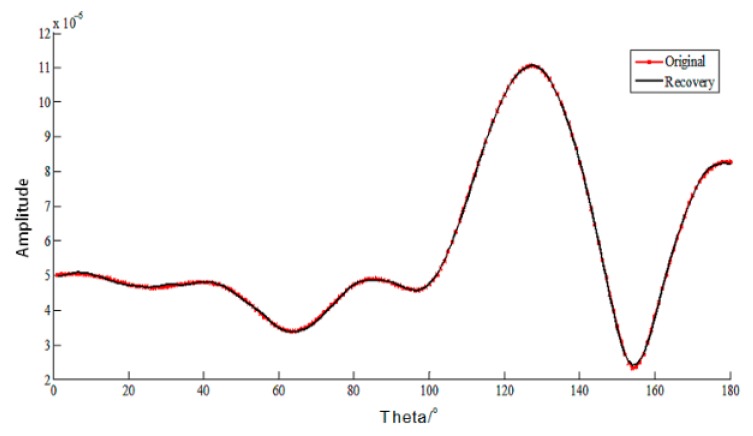
The current of PEC cube by multilevel fast multipole acceleration (MLFMA) and MLFMA with compressive sensing (CS) based on DCT basis.

**Figure 5:**
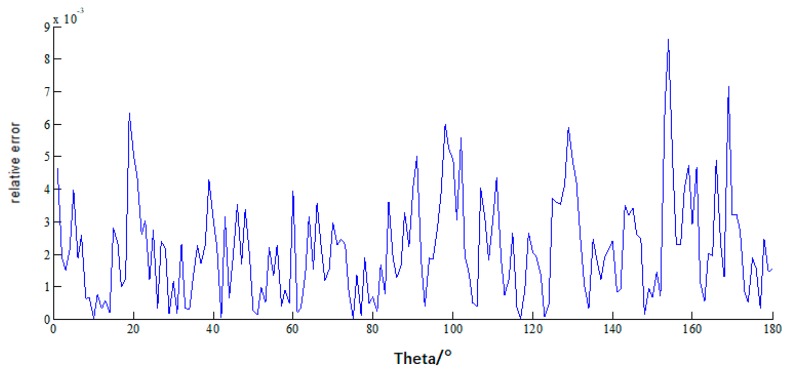
The relative error of the current of PEC cube by MLFMA and CS-MLFMA based on DCT basis.

EM parameters are set as follows: frequency is 300 MHz, the initial incident angle is $0^{o}\;$ and changed from $0^{o}$ to $180^{o}$ with the step of $1^{o}$, and CFIE is chosen with the scaling factor 0.5. For the CS theory, the measurement matrix Φ is the constructed matrix as in Equation (20), the basis matrix Ψ is set as Discrete Cosine Transform (DCT) with sparsity about 10 with respect to the corresponding basis shown in Figure 3, Orthogonal Matching Pursuit (OMP) is chosen as the reconstructed algorithm with the measuring time m=50 so the RIP can be satisfied. The original and reconstructed current and their relative error are shown in Figure 4 and Figure 5, respectively.

**Figure 6:**
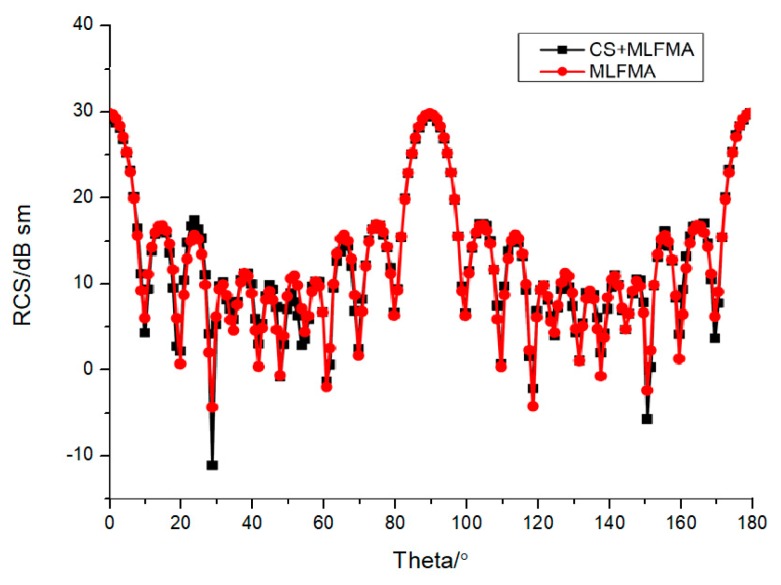
Radar cross section (RCS) for PEC cube by MLFMA and CS-MLFMA.

It can be seen the relative errors in Figure 5 are almost under $10^{-3}$, and the RCS by MLFMA and CS-MLFMA are provided in Figure 6, where the error of the reconstruction can be considered acceptable.

The computation complexity of MLFMA and CS-MLFMA is analyzed in Table 1. Even though the reconstruction is time consuming, the implementation of CS reduces the computation time while keeping the recovery valid.

**Figure 7:**
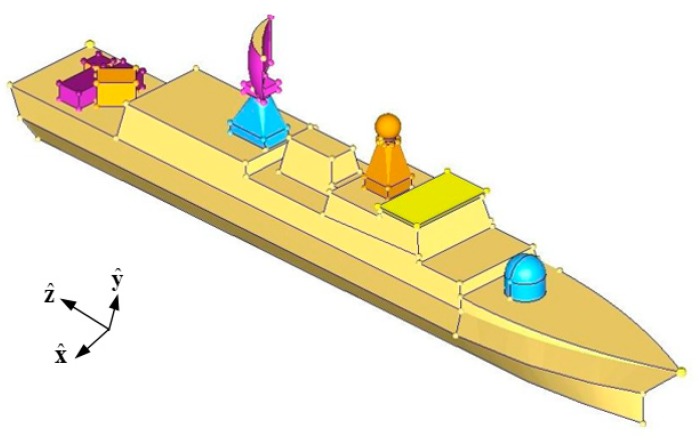
Ship model.

**Figure 8:**
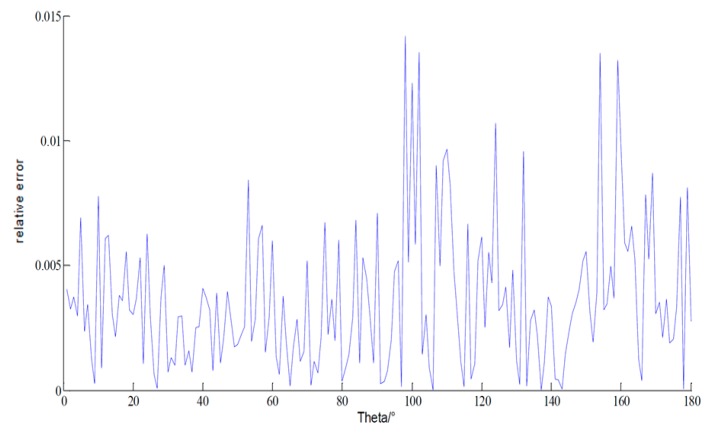
The relative error of the current of ship model by MLFMA and CS-MLFMA based on DCT basis.

For a more complex ship model in Figure 7, the simulation environment is $90^{o}$ and the measuring time m is set to 90. The relative errors between the original and reconstructed current are shown in Figure 8, which can be acceptable to expect some special points.

**Figure 9:**
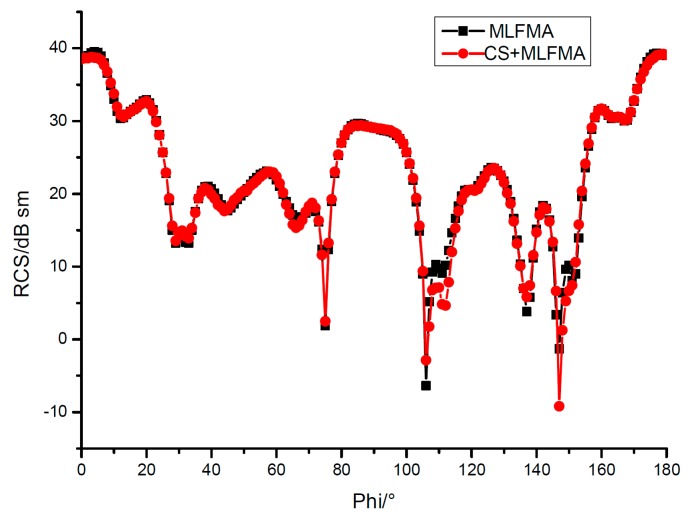
RCS for ship model by MLFMA and CS-MLFMA.

The RCS by both methods in Figure 9 show the feasibility of the CS implementation in MLFMA. Moreover, the computation time is much more improved by CS as detailed in Table 2.

### 4.2. ISAR Imaging by Using CS-MLMFA

As described in Section 3, the received data for the wide-band and multi-angle signal can be obtained by CS-MLFMA, which then can be utilized for ISAR Imaging.

**Figure 10:**
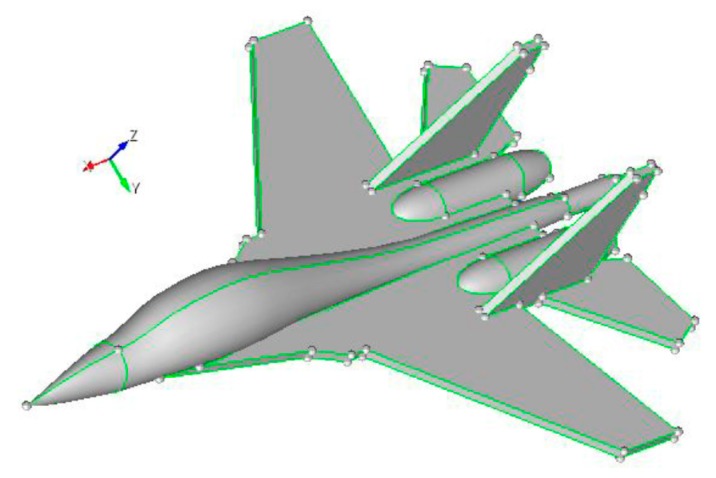
Aircraft model.

An aircraft model in Figure 10 is taken as a numerical example and the parameters are detailed in Table 3.

**Figure 11:**
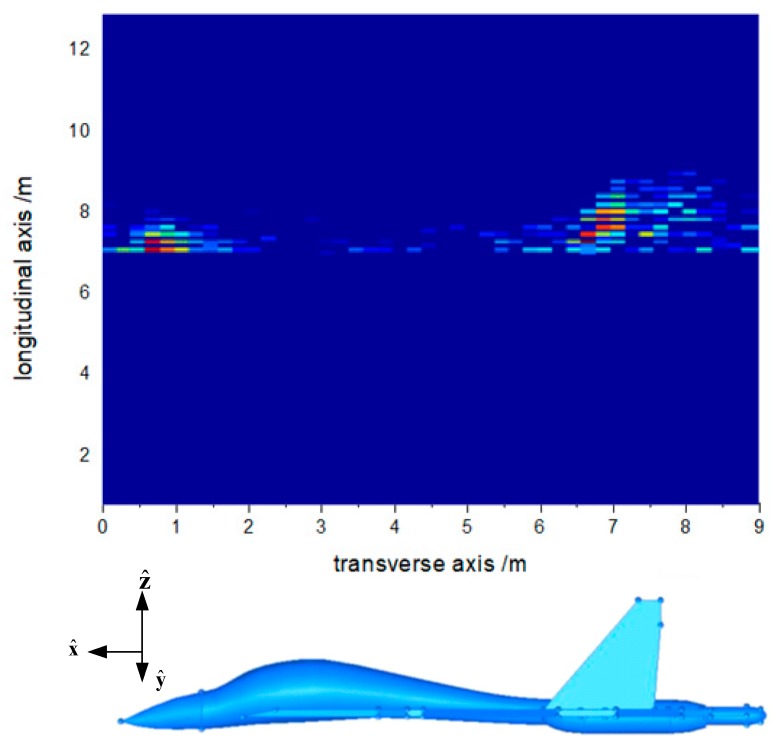
Aircraft model imaging by CS-MLFMA.

**Figure 12:**
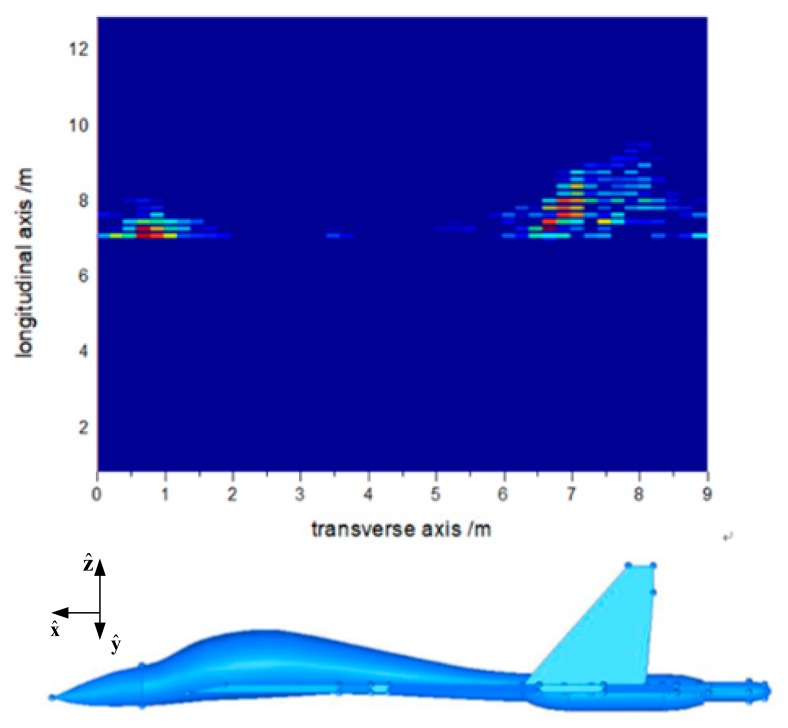
Aircraft model imaging by MLFMA.

The received data obtained by MLFMA and CS-MLFMA can be used for scattering centers extraction and then the strong scattering center imaging can be implemented as shown in Figure 11 and Figure 12, respectively.

Obviously, the ISAR imaging of the aircraft is feasible, and the computation time can be reduced by CS as in Table 4.

## 5. Conclusions

In this paper, the CS theory proposed in the information area is introduced to implement with MLFMA for a wide-angle monostatic EM scattering problem. CS-MLFMA makes a new kind of incident wave which reduces the computation complexity of repeated matrix computing. RCS obtained by CS-MLFMA is almost consistent with the data by MLFMA, which shows the approach is capable of precise estimation of the proposed method. Moreover, the received data in different incident angles at different frequencies by the new proposed CS-MLFMA are constructed as the real data for ISAR imaging, which makes the imaging more realistic. The utilization of CS in MLMFA makes the ISAR imaging successful with fewer incident waves and the computation complexity is reduced greatly, which makes the EM scattering simulation and ISAR imaging by real data meaningful.

## Figures and Tables

**Table 1:** Computation time comparison for PEC cube.

Algorithm		MLFMA	CS-MLFMA
Incident Angle		181	50
Time (min)	Iterative Inverse	7	3
	Reconstruction	0	2.9
	Total Time	8	6.9
	Improvement		13.75%

**Table 2:** Computation time comparison for ship model.

Algorithm		MLFMA	CS-MLFMA
Incident Angle		181	90
Time (min)	Iterative Inverse	2.8	2.1
	Reconstruction	0	0.8
	Total Time	3.8	2.9
	Improvement		23.70%

**Table 3:** Example parameters.

Items	Values
Body Length in *X*-axis	9
Distance (the longest wings) in *Y*-axis	6
Aircraft Length in *Z*-axis	2.4
Initial Incident Angle	74°
Angle Interval	0.5°
Number of Incident Angle	45
Frequency Range	300–1000 MHz
Frequency Interval	15 MHz
Number of Incident Frequency	65

**Table 4:** Computation time for airplane model.

Freq./GHZ	Freq. Point	CS-MLFMA/min	MLFMA/min	Improvement
[0.3, 0.4)	6	1.2	1.5	20%
[0.4, 0.5)	7	1.75	2	12.50%
[0.5, 0.6)	7	6.7	8	16.30%
[0.6, 0.7)	6	8.3	10	17%
[0.7, 0.8)	7	13	15	13.30%
[0.8, 0.9)	7	17.5	20	12.50%
[0.9, 1.0)	5	24.6	28	11.70%
**Num. of Incident Angles**			45	
